# Le syndrome de Morel-Lavallée: une entité à ne pas méconnaitre

**DOI:** 10.11604/pamj.2015.20.200.6312

**Published:** 2015-03-03

**Authors:** Ammar Mahmoudi, Ahmed Zrig

**Affiliations:** 1 Service de Chirurgie Générale et Digestive, CHU Fattouma Bourguiba de Monastir, Monastir, Tunisie; 2 Service d'Imagerie Médicale, CHU Fattouma Bourguiba de Monastir, Monastir, Tunisie

**Keywords:** Epanchement lymphatique, retard de cicatrisation, traumatisme tangentiel, Morel-Lavallée, lymphatic effusion, delayed healing, tangential trauma, Morel-Lavallée

## Image en medicine

Le syndrome de Morel-Lavallée correspond à un épanchement sérolymphatique secondaire à un traumatisme tangentiel en regard d'un tissu richement vascularisé. Les aspects morphologiques sont variables en fonction de la durée d’évolution et de l'organisation éventuelle d'une capsule fibreuse. Le traitement conservateur associe bandage compressif et ponctions-aspirations. Le traitement chirurgical doit être envisagé dans les formes résistantes. Nous rapportons l'observation d'un homme âgé de 22 ans, sans antécédents pathologiques, qui avait présenté, à la suite d'un accident de la voie publique; motocycliste heurté par une voiture où il a été trainé par terre, plusieurs plaies au niveau de l'hémi-abdomen gauche qui avaient été suturées. Le scanner abdominal a montré un foyer de lacération splénique avec intégrité du pédicule, et un hémopéritoine de faible abondance. Devant la stabilité hémodynamique on a opté pour un traitement non opératoire qui a été un succès. Trois semaines après, le patient présentait une douleur lombaire gauche. Il avait une plaie fibrineuse en regard d'un œdème douloureux lombaire gauche. La palpation trouvait une masse fluctuante sous la plaie. Il y avait un syndrome inflammatoire biologique. L’échographie des parties molles identifiait une collection liquidienne anéchogène homogène extra-aponévrotique lombaire gauche. Le diagnostic d’épanchement post-traumatique de Morel-Lavallée a été retenu. Il a été réalisé à deux reprises à dix jours d'intervalle, une ponction évacuatrice de la collection ramenant respectivement 120 cc et 70 cc de liquide séro-hématique, un pansement compressif puis une contention. Les suites étaient favorables avec amélioration de la symptomatolgie et dispariton de la collection lombaire.

**Figure 1:**
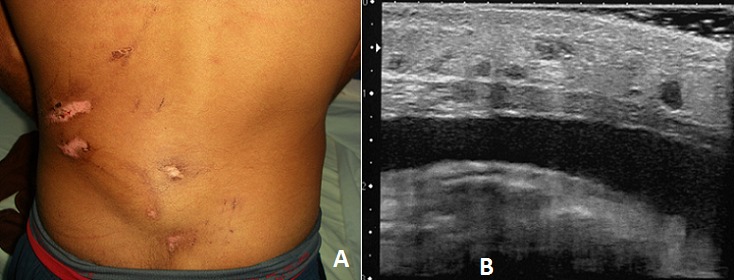
(A) œdème et plaies de la région lombaire gauche. La palpation trouvait une masse fluctuante sous les plaies; (B) l’échographie des parties molles identifiait une collection liquidienne anéchogène homogène extra-aponévrotique lombaire gauche

